# Evaluation of SARS-CoV-2 RNA shedding in clinical specimens and clinical characteristics of 10 patients with COVID-19 in Macau

**DOI:** 10.7150/ijbs.45357

**Published:** 2020-03-15

**Authors:** Iek Long Lo, Chon Fu Lio, Hou Hon Cheong, Chin Ion Lei, Tak Hong Cheong, Xu Zhong, Yakun Tian, Nin Ngan Sin

**Affiliations:** 1 Physician, Consultant, Department of respiratory medicine, Centro Hospitalar Conde de São Januário, Macau Health Bureau, Macau SAR, China; 2 Physician, Macau Academy of Medicine, Centro Hospitalar Conde de São Januário, Macau Health Bureau, Macau SAR, China; 3 Physician, Macau Academy of Medicine, Centro Hospitalar Conde de São Januário, Macau Health Bureau, Macau SAR, China; 4 Physician, Chief of service, Department of medicine, Centro Hospitalar Conde de São Januário, Macau Health Bureau, Macau SAR, China; 5 Physician, Chief of service, Department of respiratory medicine, Centro Hospitalar Conde de São Januário, Macau Health Bureau, Macau SAR, China; 6 Physician, Consultant, Department of respiratory medicine, Centro Hospitalar Conde de São Januário, Macau Health Bureau, Macau SAR, China; 7 Physician, Department of infectious diseases, Centro Hospitalar Conde de São Januário, Macau Health Bureau, Macau SAR, China; 8 Physician, Macau Academy of Medicine, Centro Hospitalar Conde de São Januário, Macau Health Bureau, Macau SAR, China

**Keywords:** novel coronavirus, SARS-CoV-2, COVID-19, qRT-PCR, Macau, nasopharyngeal swab, stool, urine, sputum

## Abstract

As a city famous for tourism, the public healthcare system of Macau SAR has been under great pressure during the outbreak of the Coronavirus Disease 2019 (COVID-19). In this study, we report clinical and microbiological features of ten COVID-19 patients enrolled in the Centro Hospitalar Conde de São Januário (CHCSJ) between January 21 to February 16, 2020. Clinical samples from all patients including nasopharyngeal swab (NPS)/sputum, urine, and feces were collected for serial virus RNA testing by standard qRT-PCR assay. In total, seven were imported cases and three were local cases. The median duration from Macau arrival to admission in imported cases was 3 days. Four patients required oxygen therapy but none of them needed machinal ventilation. No fatal cases were noted. The most common symptoms were fever (80%) and diarrhea (80%). In the "Severe" group, there was significantly more elderly patients (p=0.045), higher lactate dehydrogenase levels (p=0.002), and elevated C-Reactive protein levels compared to the "Mild to Moderate" group (p<0.001). There were positive SARS-CoV-2 RNA signals in all patients' NPS and stool specimens but negative in all urine specimens. Based on our data on SARS-CoV-2 RNA shedding in stool and the possibility of a lag in viral detection in NPS specimens, the assessment of both fecal and respiratory specimen is recommended to enhance diagnostic sensitivity, and also to aid discharge decision before the role of viral RNA shedding in stool is clarified.

## Introduction

The outbreak of 2019 novel Severe Acute Respiratory Syndrome Coronavirus 2 (SARS-CoV-2) infection or Coronavirus Disease 2019 (COVID-19) has become a global threat leading to more than 2700 deaths worldwide 1. Macau is one of the special administrative regions (SAR) located to the west of the Pearl River estuary in southern China. In 2019, more than 39.4 million visitors entered Macau. Among them, mainland tourists account for the majority, with about 28 million passengers. In addition, a large number of mainland tourists visited Macau during the Spring Festival Holiday. Hence the epidemic prevention work has been under great pressure.

The disease caused by this novel member of the coronavirus family is referred as COVID-19 by the World Health Organization (WHO) 1,2,3. The virus has spread rapidly since its recent identification in patients with severe pneumonia in Wuhan, China. As of February 27^th^, 2020, COVID-19 has been reported in 47 countries around the world and over 80,000 cases have been confirmed 1. Following the spread of the COVID-19 infection reported in Beijing, Shanghai, the US, Thailand, Republic of Korea, Japan, and Taiwan, the first confirmed case in Macau was documented on January 22^nd^, 2020, which was a case imported of a tourist from Wuhan 4. There were altogether 10 confirmed cases of COVID-19 recorded until February 16^th^, 2020 after the announcement of the first case in Macau, including seven imported cases from Wuhan and three local cases. In this retrospective study, we report clinical characteristics and microbiological features of 10 patients with COVID-19 from the Centro Hospitalar Conde de São Januário (C.H.C.S.J.) in Macau, which is a designated hospital to manage all COVID-19 patients in Macau SAR, China, and discuss what we could learn from these data.

## Methods

### Enrolment and Data collection

Between January 21^st^ and February 16^th^, 2020, we enrolled 10 patients with COVID-19 that were hospitalized at C.H.C.S.J., Macau SAR, China. The study was approved by the Hospital Medical Ethical Committee of C.H.C.S.J., which is the only public hospital in Macau that is responsible for detection, management, and follow up of patients with COVID-19. We collected data, including daily medical records and laboratory, radiological, and microbiological results, from our electronic medical system and written notes. All patients were diagnosed based on clinical symptoms and contact history with further confirmation by positive results of qualitative real-time reverse transcriptase-polymerase chain reaction assay (qRT-PCR) targeting the ORF1ab/N gene (BioGerm, China) in respiratory specimens according to WHO interim guidance.

### Severity classifications

Patients' severities were classified based on the “Guideline on the management of COVID-19, version 6” published by National Health Commission of the People's Republic of China 5. "Mild" was defined as mild clinical symptoms or asymptomatic with no signs of pneumonia in imaging; "Moderate" was defined as having fever or respiratory tract symptoms and signs of pneumonia in imaging; "Severe" was classified if one of the following was present: a) dyspnea with a respiratory rate of ≥ 30 per minute, b) saturation ≤ 93%, and c) PaO2 / FiO2 ≤ 300mmHg; "Critical" was classified if one of the following was present: a) respiratory failure requiring mechanical ventilation, b) shock, and c) co-existing multiple organ failure requiring close monitoring in the Intensive Care Unit (ICU).

### Real-Time Reverse Transcriptase-Polymerase Chain Reaction Assay for SARS-CoV-2

Samples were taken from different specimens, including nasopharyngeal swabs (NPS), sputum, urine, and stool in all patients where available. The extraction of nucleic acid from the respiratory, urine, or stool samples was performed using EasyMag in accordance with the manufacturer's instructions (bioMerieux, France). Extracted nucleic acid samples were tested for SARS-CoV-2 with qRT-PCR using a commercial SARS-CoV-2 (previously known as 2019- nCoV) ORF1ab/N Gene Nucleic acid detection kit (BioGerm, China) and the LightCycler 480 real-time PCR system (Roche, Switzerland) in accordance with manufacturer's instructions; 5 μL of extracted RNA were added into 20 μL of the reaction mixture. Each 20 μL reaction mixture contained 12 μL of qRT-PCR reactant, 4 μL of qRT-PCR enzyme Mix, and 4 μL primer probe wHCoV (ORF1ab/N). Reactions were incubated at 50°C for 10 min and 95°C for 5 min followed by 40 cycles at 95°C for 10 s and 55°C for 40s. Then, the samples were subjected to melting curve analyses (95°C for 5 s and 65°C for 1 min followed by a gradual increase in temperature to 97°C with continuous recording of fluorescence). The results were interpreted according to the kit manual. A cycle threshold value (Ct-value) less than or equal to 35 was defined as a positive test result, and a Ct-value of more than 38 was defined as a negative test result. A medium load, defined as a Ct-value of 36 to 38, required confirmation by retesting and was reported as inconclusive.

### Statistical Analysis

Statistical analyses were conducted using SPSS ver. 18.0 (SPSS Inc., Chicago, IL, USA). Continuous data were presented as mean ± standard deviation (SD), and dichotomous variables were presented as percentages. Unpaired Student's t-tests and Chi- squared tests were used to compare the clinical and laboratory characteristics of the “Mild-Moderate” and “Severe” groups. If continuous data did not follow normal distributions, Mann-Whitney U-tests would have been performed. In all comparisons, a p-value of <0.05 was considered statistically significant.

## Results

### Epidemiological Characteristics

**Figure 1:**
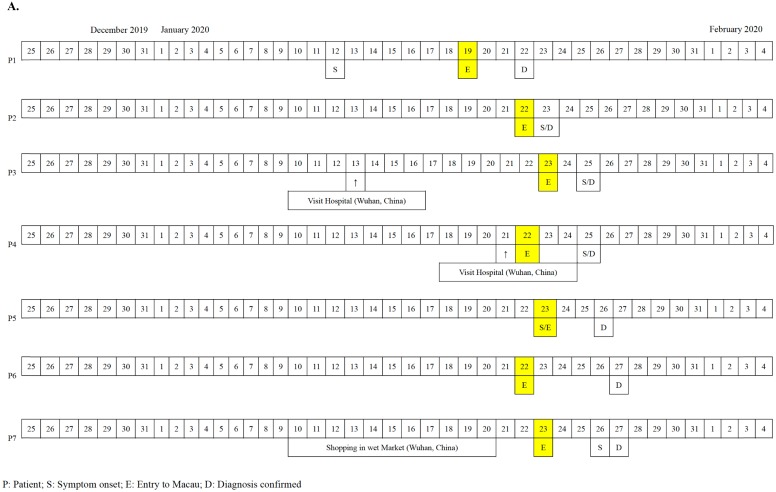

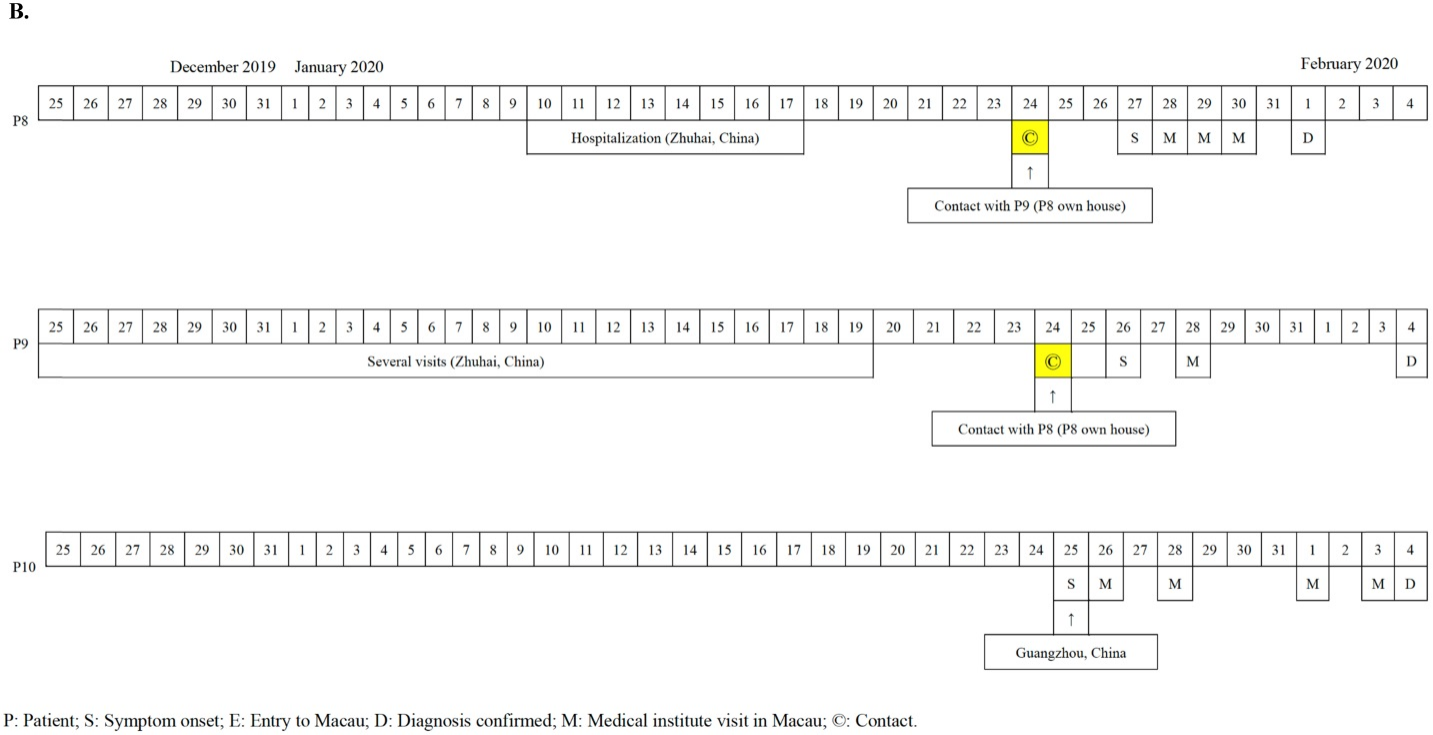
** A.** Chronology of 7 imported cases of COVID-19 in Macau. **B.** Chronology of 3 local cases of COVID-19 in Macau.

The first seven cases of COVID-19 in Macau were imported cases from Wuhan diagnosed from the 22^nd^ to 27^th^ of January, 2020 (Figure 1A). The patients' documented date of entry to Macau ranged from January 19^th^ to 23^rd^, 2020. All seven patients (70%) were residents of Wuhan, China and had travelled to Macau for sightseeing. Patients 4 and 6 have a mother-child relationship. All seven Wuhan patients denied any Huanan seafood wholesale market visits or intake of gaming meat. Patients 3 and 4 visited a hospital in Wuhan 12 and 4 days prior to admission, respectively. The 8^th^, 9^th^, and 10^th^ cases were Macau residents (Figure 1B). All of them had a travel history to cities of Guangdong province in January, 2020 but denied travelling to any cities in Hubei Province. Notably, patient 8 was once admitted to a hospital in Zhuhai City due to herpes zoster from January 10^th^ to 17^th^, 2020. Provided with the aunt-niece relationship between patient 8 and patient 9, the suspicious contact history between them was a family gathering in the house owned by patient 8 eight days before her diagnosis. No medical staff in our hospital were infected with SARS-CoV-2 during the study period.

### Clinical Characteristics

This report includes 10 hospitalized patients with confirmed COVID-19. All patients (100%) were admitted to the isolation ward, and none of them required ICU transfer. Their demographic and clinical information are summarized in Table 1. The median age of the patients was 54 years old (IQR:27-64). Three patients were male (30%) and one was a teenager (age 15 years) (Table 1). For the seven imported cases, the median duration from Macau entry to hospital admission was three days (IQR:2-4). Of these 10 patients, five (50%) had one or more coexisting medical conditions, such as hypertension, dyslipidemia and hepatitis B infection. The most common symptoms were fever (80%) and diarrhea (80%) followed by coughing (50%), dyspnea (50%), sore throats (50%), nausea (50%) and myalgia (30%). Less common symptoms included rhinorrhea, nasal congestion, dizziness, and abdominal pain. One patient (patient 6) remained asymptomatic during the whole course. In our cohort, there were 20% mild, 40% moderate, and 40% severe cases based on the criteria stated above. No patient was categorized as “Critical.” The MuLBSTA score 6, which is a model proposed in 2019 for predicting mortality in viral pneumonia and consists of six indices including multi-lobular infiltration, lymphopenia, bacterial co-infection, smoking history, hypertension and age, was calculated. All scores from our cases were less than 11 which predicted a low risk of mortality (median:5, range:0-9) and it was coherent to the severity of our cases that we had no patients categorized as “Critical”.

### Laboratory Parameters

**Figure 2:**
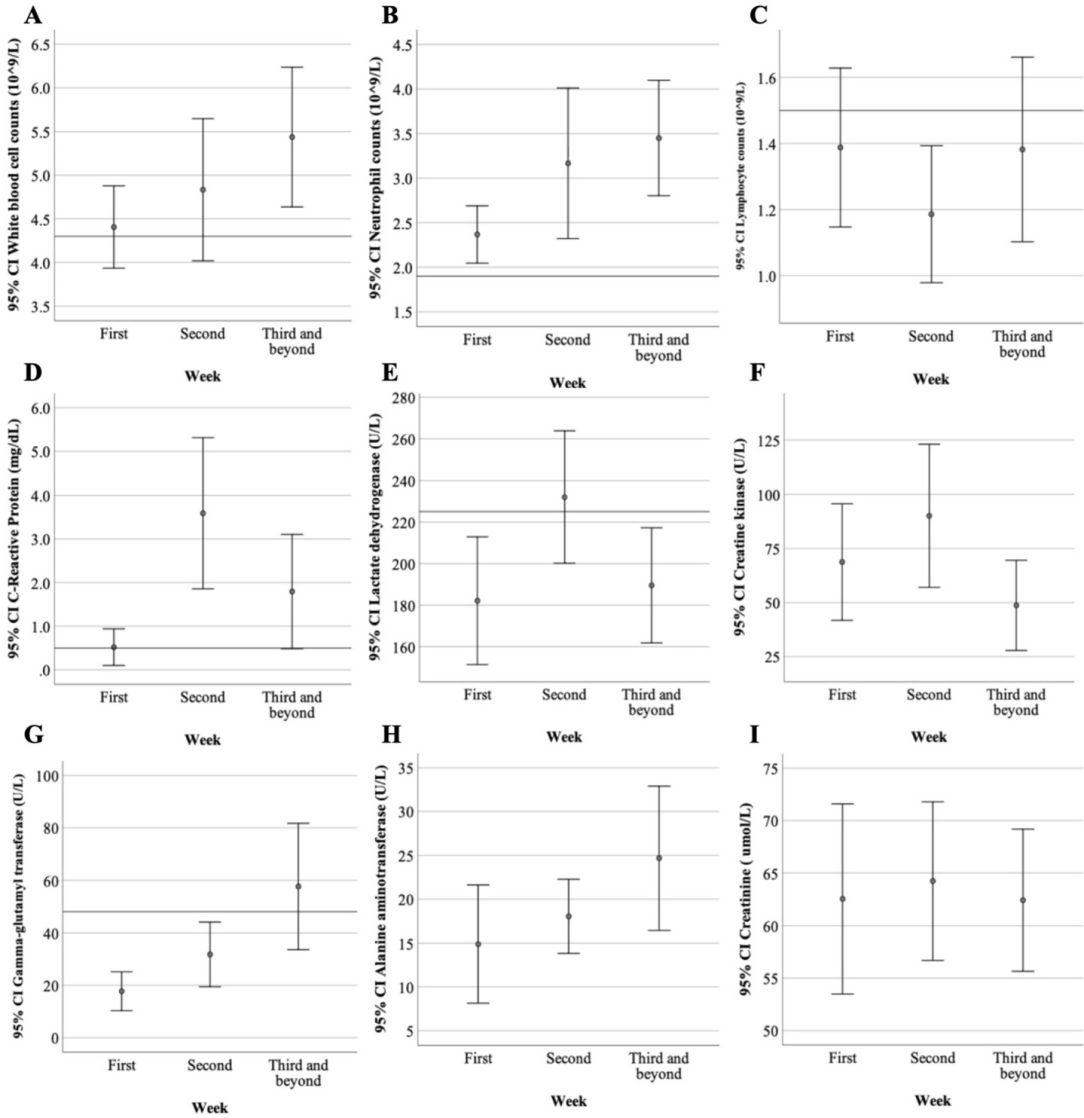
** The dynamic change and evolution in laboratory data of ten COVID-19 patients from the first week to the third week and beyond since symptoms of onset. A.** The lower bound of mean white blood cell counts was back to normal two weeks after symptoms onset. **B.** Neutrophil counts were gradually increased from the first to the third week. **C.** Lymphocyte counts reached its lowest levels in the second week with the elevation of **D.** C-Reactive Protein, **E.** lactate dehydrogenase and **F.** creatine kinase suggesting clinical exacerbation. Elevating **G.** Gamma-glutamyl transferase and **H.** Alanine aminotransferase levels were noted which might be related to viral infection per se or medication side effects. **I.** The creatinine level was remained steady during the study period.

Laboratory studies, including hemograms and biochemical and inflammatory markers within 24 hours of admission are summarized (Table 2). Leukopenia was noted in only 20% of patients (median 5.0, IQR:4.4-5.4) while lymphopenia was noted in 70% of patients (median 1.3, IQR:1.0-1.7). More than half of the patients (60%) presented with elevated lactate dehydrogenase (LDH) levels (median 228, IQR:206.5-240.5). Only one patient (10%) had elevated creatine kinase (CK) levels. Elevated C-reactive protein (CRP) was noted in 40% of patients (median 0.4, IQR:0.1-0.7). Procalcitonin levels were within the normal range in all patients. The dynamic change and evolution in the laboratory data of COVID-19 patients during hospitalization were demonstrated (Figure 2). Lymphopenia and elevated CRP and CK levels peaked in the second week after symptom onset, thus possibly indicating disease progression. After the third week of symptoms onset, we noted that white blood cells, such as neutrophils and lymphocytes, increased alongside the downtrend of CK, LDH, and CRP levels; this suggests a stable phase in the disease progression. Patients categorized as “Severe” had different laboratory features compared to those who were “Mild to Moderate” (Table 3). In the "Severe" group, there was significantly more elderly patients (p=0.045), higher LDH levels (p=0.002), and elevated CRP levels compared to the "Mild to Moderate" group (p<0.001).

### Radiological Features

**Figure 3:**
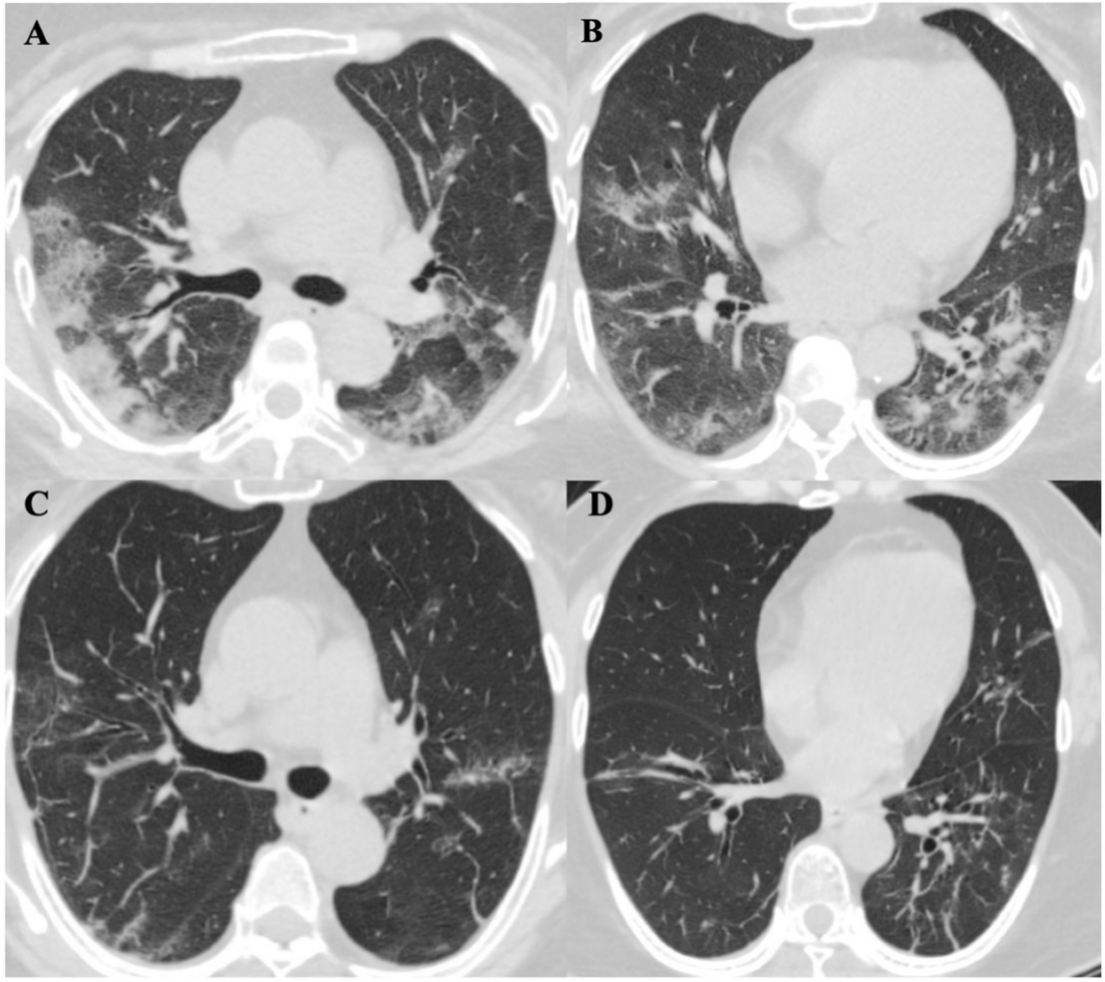
** ChestComputed Tomographic Images of a 57-year-old patient (Patient 5) with SARS-CoV-2 pneumonia during hospitalization. A. and B.** were two different levels of Chest CT images in patient 5 on day 9 of symptoms onset, it showed bilateral distribution of patchy shadows and ground glass opacity. **C. and D.** showed significant lesions absorption on day 21 of symptoms onset comparing to the same level images of A. and B.

Chest computed tomography (CT) scans were performed for all patients on the day of diagnosis. Initially, six patients (60%) were noted to have a bilateral distribution of patchy shadows or ground glass opacity except in four patients (patients 2, 4, 6, and 7) (Table 4). Patients received serial CT scans in the follow up or according to clinical condition changes afterwards. Patient 2 was found to have multiple patchy ground-glass opacities in both lungs in a CT scan on day four after admission, and patient 7 presented with multiple patchy lesions in bilateral lungs day 10 after admission. In contrast, there were two patients (20%) (patients 4 and 6) who had no radiological evidence of pneumonia during hospitalization. In total, eight (80%) patients showed bilateral pneumonia (100%). No pneumothorax or pleural effusion occurred in any patient. The evolution of CT images in one patient (patient 5) is illustrated in Figure 3, which shows the serial change of lung lesions.

### Management and Prognosis

Oral antiviral treatments with protease inhibitors of lopinavir and ritonavir tablets (400 mg/100mg) were administered twice daily to all (100%) patients (Table 4). The duration of antiviral treatment was aimed to beat least 14 days or longer depending on clinical and laboratory parameters such as positivity of nucleic acid. Interferon-beta-1b (250mcg) was administered every other day to three patients (one moderate case and two severe cases). All patients were administered with antibiotics treatment: seven (70%) patients were treated with single antibiotics and three (30%) patients with combination therapy. The antibiotics, including cephalosporins, quinolones, and macrolides, generally covered common pathogens and atypical pathogens. Three (30%) severe cases were treated with intravenous methylprednisolone sodium succinate (about1mg/kg/day) for 3 to 5 days (median 4). All four (100%) patients required oxygen supplements through a nasal cannula over the course of treatment. None of them required oxygen support higher than nasal cannula nor any mechanical ventilation. No patient presented with acute respiratory distress syndrome (ARDS). By the end of February 16^th^, five (50%) patients, including one severe case, had been discharged, and five (50%) patients were still in hospital. Patients were discharged if their conditions fulfilled the following criteria: afebrile, improved respiratory symptoms and imaging studies, consecutive two respiratory specimens tested negative by qRT-PCR taken at least 24 hours apart, and clinical eligibility evaluated by a pneumologist. No fatal cases were noted.

### Microbiological Panels

All 10 patients were diagnosed through detected RNA signals in respiratory specimens as follow: 90% by nasopharyngeal swab (NPS) and 10% by sputum. Serial qRT-PCR for SARS-CoV-2 were performed for different specimens, including NPS, urine, and stool from these 10 patients (Table 5). There were positive SARS-CoV-2 RNA signals in all patients' NPS (100%) and stool specimens (100%) but negative in all urine specimens (0%). The average viral RNA conversion time in both NPS and feces were 18.2 days (SD:4.6) and 19.3 days (SD:3.4), respectively. The evolution pattern for the qRT-PCR results of all 10 cases are summarized in Table 6.

## Discussion

Common laboratory abnormalities in COVID-19 patients include decreased white blood cell, lymphocyte, and platelet counts, and an increased LDH, CK, and CRP levels 7,8,9,10,11. Suspected cytokine storms and a series of immune responses induced by SARS-CoV-2 have also been suggested 11. Our data echoed the same findings of lymphopenia and elevated LDH levels during the second week after symptom onset. Major complications, including arrhythmias, shock, ARDS, and multiple organ failure were not present. Less than half of patients (40%) required low-flow oxygen therapy, and none of the patients needed invasive ventilation or extracorporeal membrane oxygenation. In this cohort, no patients were admitted to the ICU or died; 50% were discharged, and 50% remained hospitalized. For those who were discharged (n = 5), the median hospital stay was 20 days (IQR:19-21). We believe that early diagnosis and adequate supportive treatment may have positive effects on stabilizing the patients. Noticeably, the median duration from Macau arrival to admission was only 3 days, that early detection of imported cases may be contributed to the effective quarantine policy organized by the Macau government, and may minimize the spread of local outbreak in Macau as no new diagnosed cases from February 4^th^ to 27^th^,2020 1.

About 75% of cases of COVID-19 have been reported with bilateral pneumonia 11, while bilateral pneumonia was noted in 80% of our cases. The primary findings in CT images of COVID-19 patients were as follow 7,12 : ground glass opacities (GGO), inter- or intra-lobular septal thickening, and air space consolidation. Lung abnormalities on chest CT images were reported to show the greatest severity approximately 10 days after the initial onset of symptoms while consolidation could increase up to two weeks and be gradually absorbed 12. Fibrous stripes were recognized upon improvement in the disease course 13. These features were mostly in line with our cases (Figure 3). Notably, a few slightly enlarged lymph nodes were found in the mediastinum in two of the 10 cases (patients 5 and 7), which the enlargement subsided in the fourth CT performed on day 21 after symptom onset in patient 5. Reactive lymphadenopathy due to infection might be one of the explanations.

Currently, there is no medications for COVID-19 approved by FDA. Antiviral therapies such as ritonavir and lopinavir, with or without ribavirin, have shown some success in the treatment of SARS 14, and an early report suggests that it might have similar efficacy in the treatment of COVID-19 15. Meanwhile, phase III trials of remdesivir, ritonavir and lopinavir had been initiated 16. In this cohort, all our patients received lopinavir/ritonavir. Although it is impossible to draw any conclusion of its efficacy before having the results from the trials, the side effects were well tolerated to all patients and the empirical use may be justified under the circumstances of epidemic. Although the use of corticosteroids is controversial 17, three patients had received methylprednisolone for a short duration due to clinical and radiological exacerbations. We believe that this is still advisable for patients who have severe illness, based on the hypothesis of reducing cytokine storm and inflammation-mediated lung injury. The settlement of this debate still requires further high-quality studies to justify or against the use of corticosteroids, and also to determine the optimal timing, dosage and duration of administration.

NPS remains a mainstay sampling method for diagnosing SARS-CoV-2 infections; it is especially supported by recent data on SARS-CoV-2 viral loads in upper respiratory specimens with higher viral loads detected in the nasal area than in the throat 18. Of our cases, 90% were diagnosed by RNA signals in NPS specimens. Interestingly, patient 5 was diagnosed via the detection of viral RNA in sputum after one negative and one inconclusive result from NPS specimens. All five NPS specimens taken six days after symptom onset remained negative or inconclusive by qRT-PCR in patient 5. In contrast, fecal samples were subsequently collected and virus RNA were detectable in 90% of our patients from their first sample by qRT-PCR. In addition, in patient 1, SARS-CoV-2 RNA was detected in feces till 14 days after the onset of symptoms. This echoes the result from the first case in the U.S. that featured positive virus RNA in the stool 9, but is in contrast to a case series reported by Chan *et al* 8. A recent study has shown that cell receptor angiotensin converting enzyme II was also expressed in absorptive enterocytes in the ileum and colon 19. Based on our data on viral shedding in stool and the possibility of a lag in viral detection in NPS specimens, it is not unreasonable to perform additional testing of both stool and respiratory specimens to enhance the detection sensitivity and minimize the "escape" of infected individuals.

Although virus RNA shedding in feces was common in our patients, the question of whether it is a potential transmission route of SARS-CoV-2 deserves further evaluation since the viability of SAR-CoV-2 in feces is uncertain. Thus, we advocate that we should consider a combined assessment of both fecal and respiratory specimens for all patients during hospitalization. Furthermore, it is worth questioning the current discharge policy which is based on the clinical, radiological condition and twice negative results from qRT-PCR in respiratory specimens 5. Our data suggested some patients may have the delay of virus RNA conversion in the stool sample. Patients with negative NPS and positive stool samples may be undergoing the convalescent phase and their infectivity during this phase remained unknown. We believed that the discharge of those patients should require additional considerations. For example, transferring those with low medical demands from hospital to another isolation facilities or extending the isolation period at home with strict infection control and follow-up may be considered before more data available against this transmission route, especially when healthcare resource is in scarcity during the outbreak. Notably, some recovered patients were reported to have virus RNA reappeared in respiratory samples after discharge 20. We believed that false-negative RT-PCR test results, virus RNA residues or/and re-activation may be contributed to this phenomenon. Whether these patients are contagious or not, we should take a more cautious approach unless proven otherwise. Serial qRT-PCR tests and home isolation for discharged patients could provide a safety net to block the potential transmission. In our cohort, 70% of cases were imported that it was difficult to follow their conditions when they returned home in Mainland China, but they were advised to be followed in local medical institutions.

A report suggested that 10% of patients present with diarrhea and nausea initially 7. Another study of 1099 patients in China found that nausea or vomiting (5.0%) and diarrhea (3.8%) were uncommon 21. While our data was contrary to these reports that diarrhea (80%) and nausea (50%) were more common in this cohort. The exact reason for these discrepancies has not been identified yet. However, given the fact that virus RNA was found in all our patients' stool specimens and they had fewer respiratory symptoms with none of them using mechanical ventilation, there may be two different subtypes of COVID-19 manifestations as “gut-tropism” and “lung-tropism” speculatively. However, the impact of other confounding factors such as side effects of antiviral or antibiotics may need to be considered. Further studies on the impact of individual genetic variability in response to the virus may reveal a clearer picture of this issue.

During the SARS outbreak, a report stated that three hospital cleaners were infected by possible fomite transmissions without direct patient contact 22. Under these circumstances, measures including strict precautions, adequate protective devices, and infection control training should be implemented for all hospital workers, especially assistants and cleaners who handle the excreta of these patients and toilet disinfection. Moreover, public education on toilet hygiene, such as closing the toilet lid before flushing, avoiding low water levels of sewage U-traps, and reiterating the importance of hand hygiene, may minimize the risk of community outbreaks 23. Adequate infection control training for all medical staff and provision of personal protection equipment such as masks, face shields, goggles, and protective clothes are recommended by the WHO 24. Nevertheless, the Macau Health Bureau played a crucial role in coordinating all parties to ensure sufficient logistic support that achieved most of the recommendations from WHO during the outbreak of SARS-CoV-2. Although C.H.C.S.J is the only hospital designated for COVID-19 patients in Macau, no in-hospital transmission has been noted so far.

This study has several limitations. First, the number of patients in this study was relatively small and as such, it is hard to draw a definite conclusion. Second, evaluation of viremia from patient's serum was not available in this study. Third, virus isolation from different specimens was not performed. Hence, it was impossible to determine whether RNA shedding in specimens correlated to the virus's viability. Finally, half of the enrolled patients are still hospitalized at the time of the submission of this paper. Therefore, there may have been bias regarding the prognosis of the patients.

## Conclusion

Neither in-hospital transmission nor large-scale community transmission occurred during the study period. An average of 3 days was taken to detect imported cases by Macau SAR. Based on our data on SARS-CoV-2 RNA shedding in stool and the possibility of a lag in viral detection in NPS specimens, the assessment of both fecal and respiratory specimen is recommended to enhance diagnostic sensitivity, and also to aid discharge decision before the role of viral RNA shedding in stool is clarified.

## Figures and Tables

**Table 1:** Summary of demographic and clinical information of ten patients with SARS-CoV-2 infection in Macau from Jan 21 to Feb 16, 2020

Age (Median, IQR)	54 (27 - 64)
<18 years old (N, %)	1 (10%)
18 to 59 years old (N, %)	6 (60%)
≥60 years old (N, %)	3 (30%)
**Gender (Male, N, %)**	3 (30%)
**Comorbidity (N, %)**	
Hypertension	3 (30%)
Dyslipidemia	2 (20%)
Past Hepatitis B infection	1 (10%)
**Current smoker (N, %)**	0 (0%)
Ex-smoker	2 (20%)
**Visited local hospital in Wuhan, China (N, %)**	2 (20%)
**Visited local hospital in Zhuhai, China (N, %)**	1 (10%)
**Visited Huanan seafood wholesale market or ate game meats**	0 (0%)
**Interval between symptom onset and diagnosis (Days, median, range)**	5 (1 - 11)*
**Interval between Macau arrival and diagnosis in seven imported cases (Days, median, range)**	3 (1 - 5)
**Symptoms and signs (N, %)**	
Body Temperature ≥ 37.5℃	8 (80%)
Diarrhea (≥3 times/day)	8 (80%)
Cough	5 (50%)
Dyspnea	5 (50%)
Sore throat	5 (50%)
Nausea	5 (50%)
Myalgia	3 (30%)
Rhinorrhea	2 (20%)
Nasal congestion	2 (20%)
Dizziness	2 (20%)
Abdominal pain	2 (20%)
**Severity classification^¶^(N, %)**	
Mild	2 (20%)
Moderate	4 (40%)
Severe	4 (40%)
Critical	0 (0%)
**MuLBSTA score (Median, range)^#^**	5 (0 - 9)
**Discharged patients (N, %)**	5 (50%)
Day of hospital stay (Days, median, IQR)	20 (19-21)

*Patient 6 did not present any symptoms before admission.¶Classification based on “Guideline on the management of COVID-19, version 6” published by National Health Commission of the People's Republic of China. ^#^In-hospital mortality prediction of viral pneumonia: MuLBSTA score: 0-11 = Low risk; ≧12 = High risk

**Table 2:** The initial laboratory features of ten patients with COVID-19on admission

	Normal Range	Median (IQR) (Total N = 10)
White blood cell count, x 10^9^/L	4.3-10.0	5.0 (4.4-5.4)
Leukopenia (N,%)	2 (20%)	
Neutrophil count, x 10^9^/L	1.9-7.3	2.6 (2.3-3.6)
Neutropenia (N,%)	1 (10%)	
Lymphocyte count, x 10^9^/L	1.5-4.0	1.3 (1.0-1.7)
Lymphopenia (N,%)	7 (70%)	
Monocyte count, x 10^9^/L	0.2-0.9	0.5 (0.5-0.6)
Hemoglobin, g/dL	12.0-16.0	13.1 (12.5-14.0)
Platelet count, x 10^9^/L	100-400	178 (163.5-206.8)
Prothrombin time, sec.	12.2	12.2 (11.7-12.8)
Activated partial thromboplastin time, sec.	30.0	34.5 (32.4-37.2)
Creatine kinase (CK), U/L	<190	87 (60.8-138.5)
Elevated CK (N,%)	1 (10%)	
Lactate dehydrogenase (LDH), U/L	135 -225	228 (206.5-240.5)
Elevated LDH (N,%)	6 (60%)	
Aspartate aminotransferase(AST), U/L	<= 41	20 (9.0-42.5)
Alanine aminotransferase (ALT), U/L	<= 40	24 (19.3-35.3)
Elevated AST or ALT (N,%)	2 (20%)	
Total bilirubin, umol/L	0 - 24	6 (4.0-7.0)
Urea, mmol/L	2.9 - 8.2	3.8 (3.0-4.3)
Creatinine, umol/L	59 - 104	56 (46.3-70.8)
C-Reactive Protein (CRP), mg/dL	< 0.5	0.4 (0.1-0.7)
Elevated CRP (N,%)	4 (40%)	
Procalcitonin, ng/mL	< 0.06	0.03 (0.03-0.04)

**Table 3:** Comparison of clinical and laboratory data between COVID-19 patients with mild-moderate and severe disease severity

	Disease severity		
	Mild to Moderate (N=6)	Severe (N=4)	*P* value
**Age (Mean ± SD)**	37± 19	61± 5	.045
**Male (N, %)**	2 (33%)	1 (25%)	.778
**Comorbidity (N, %)**	1 (17%)	2 (50%)	.260
**Ex-smoker (N, %)**	1 (17%)	1 (25%)	.747
**Laboratory data during hospitalization (Mean ± SD)**			
Creatinine, umol/L	66±15	58±16	.038
Aspartate aminotransferase, U/L	19± 8	24± 9	.031
Lactate dehydrogenase, U/L	183± 61	238 ± 52	.002
White blood cell, x 10^9^/L	4.51± 1.18	5.57± 1.99	.014
Lymphocyte, x 10^9^/L	1.40 ± 0.54	1.18 ± 0.49	.116
Neutrophil, x 10^9^/L	2.49 ± 0.90	3.82 ± 1.81	.002
C-Reactive Protein (CRP), mg/dL	1.65 ± 3.16	2.63 ± 3.02	.231
Elevated CRP (N/total sample, %)	12/35 (34%)	22/25 (88%)	<.001

**Table 4:** Summary of radiological features and treatment of ten patients with SARS-CoV-2 infection according to the disease severity in Macau from Jan 21 to Feb 16, 2020

		Disease severity		
	Total (N = 10)	Mild (N = 2)	Moderate (N = 4)	Severe (N = 4)
**Imaging Findings (N, %)**				
Negative	2 (20%)	2 (100%)	0 (0%)	0 (0%)
Unilateral pneumonia	0 (0%)	0 (0%)	0 (0%)	0 (0%)
Bilateral pneumonia	8 (80%)	0 (0%)	4 (100%)	4 (100%)
**Treatment (N, %)**				
Antiviral therapy	10 (100%)	2 (100%)	4 (100%)	4 (100%)
Interferon therapy	3 (30%)	0 (0%)	1 (25%)	2 (50%)
Glucocorticoid therapy (Methylprednisolone)	3 (30%)	0 (0%)	0 (0%)	3 (75%)
Antibiotics	10 (100%)	2 (100%)	4 (100%)	4 (100%)
Oxygen therapy	4 (40%)	0 (0%)	0 (0%)	4 (100%)
Mechanical ventilation	0 (0%)	0 (0%)	0 (0%)	0 (0%)

**Table 5:** Summary of microbiological panels of ten patients with confirmed SARS-CoV-2 infection admitted to our hospital in Macau from Jan 21 to Feb 16, 2020

Real-time PCR (ORF1ab/N gene)*	Patient 1		Patient 2	Patient 3	Patient 4	Patient 5	Patient 6	Patient 7	Patient 8	Patient 9	Patient 10	Total
Nasopharyngeal swab(NPS)	5/8		10/13	6/10 (1 inconclusive)	6/8	3/10 (2 inconclusive)	9/12	5/7 (1 inconclusive)	5/6 (1 inconclusive)	6/7	2/3 (1 inconclusive)	57/84 (68%)
Sputum	NA		NA	NA	NA	1/1	NA	NA	NA	NA	NA	1/1(100%)
Urine	0/4		0/6	0/5	0/6	0/6	0/5	0/4	0/5	0/5	0/3	0/49(0%)
Stool	1/8		7/10	4/6	3/3	5/8 (1 inconclusive)	4/6	5/8 (1 inconclusive)	6/8	10/10	1/12 (9 inconclusive)	46/79 (58%)
**Average viral RNA conversion time (days, mean, standard deviation)**												
Nasopharyngeal swab		18.2 (4.6)										
Feces		19.3 (3.4)										
												

*Results = number of specimens detected / total number of specimens performed during the course NA: Not available; Inconclusive: Ct-value of 36 to 38.

**Table 6:** Evolution pattern of SARS-CoV-2 RNA positivity among ten patients with COVID-19 during hospitalization

	Sample	Admission day																							
		1	2	3	4	5	6	7	8	9	10	11	12	13	14	15	16	17	18	19	20	21	22	23	24
**Patient 1**	NPS	■		■			■	■	△		■			△			△								
	Urine			△			△			△						△									
	Feces			■			△	△	△	△	△	△				△									
**Patient 2**	NPS	■				■	■	■		■			■			■		■	△	■			△		■
	Urine		△			△		△	△						△				△						
	Feces		■			■	■		■	■	■			△						■		△		△	
**Patient 3**	NPS	■		■	■			■			◎			△	■	■		△							
	Urine		△			△	△	△								△									
	Feces			■	■				■			■						△		△					
**Patient 4**	NPS	■		■	■	■			■			△			△										
	Urine		△			△		△				△		△		△									
	Feces														■		■	■							
**Patient 5**	NPS	△		◎	◎	△	△			■		■		■					△		△				
	Urine					△		△	△					△			△	△							
	Feces					■	■	■	◎	■						■	△	△							
	Sputum			■																					
**Patient 6**	NPS	■		■	△		■			■			■		■	■	■		■	△					
	Urine				△		△	△					△		△										
	Feces								■	■				■		■				△					
**Patient 7**	NPS	■				■		△	■			■					■								
	Urine			△	△			△						△											
	Feces			■		■			◎					△		■		■							
**Patient 8**	NPS	■		■		■		■			■														
	Urine			△	△	△	△		△																
	Feces			■		■		■	■	■	■		△												
**Patient 9**	NPS	■			■			△		■		■	■												
	Urine		△	△	△	△	△																		
	Feces		■	■	■	■	■		■		■	■	■		■										
**Patient 10**	NPS	■								◎															
	Urine		△		△	△																			
	Feces		◎	◎	◎	◎	◎	◎	◎	◎	△	■													

Abbreviations: NPS, nasopharyngeal swab; ■: Positive;△: Negative; ◎: Inconclusive.
